# Glycosaminoglycan-Based Cryogels as Scaffolds for Cell Cultivation and Tissue Regeneration

**DOI:** 10.3390/molecules26185597

**Published:** 2021-09-15

**Authors:** Annika Wartenberg, Jürgen Weisser, Matthias Schnabelrauch

**Affiliations:** Biomaterials Department, INNOVENT e.V., Pruessingstrasse 27B, 07745 Jena, Germany; jw1@innovent-jena.de

**Keywords:** cryogels, cell scaffolds, tissue engineering, glycosaminoglycans, hyaluronan, chondroitin sulfate, heparin, drug delivery

## Abstract

Cryogels are a class of macroporous, interconnective hydrogels polymerized at sub-zero temperatures forming mechanically robust, elastic networks. In this review, latest advances of cryogels containing mainly glycosaminoglycans (GAGs) or composites of GAGs and other natural or synthetic polymers are presented. Cryogels produced in this way correspond to the native extracellular matrix (ECM) in terms of both composition and molecular structure. Due to their specific structural feature and in addition to an excellent biocompatibility, GAG-based cryogels have several advantages over traditional GAG-hydrogels. This includes macroporous, interconnective pore structure, robust, elastic, and shape-memory-like mechanical behavior, as well as injectability for many GAG-based cryogels. After addressing the cryogelation process, the fabrication of GAG-based cryogels and known principles of GAG monomer crosslinking are discussed. Finally, an overview of specific GAG-based cryogels in biomedicine, mainly as polymeric scaffold material in tissue regeneration and tissue engineering-related controlled release of bioactive molecules and cells, is provided.

## 1. Introduction

Cryogels are three-dimensional, sponge-like network structures with interconnecting pores [1,2,3,4,5]. The gel-like structures are commonly synthesized by a crosslinking process from reactive monomers and/or polymeric precursors at sub-zero temperatures. While the crosslinking process takes place, ice crystals, formed from the liquid phase, acting as pore-forming agents (porogens). As a result of this process, a material architecture quite similar to the native structure of the extracellular matrix can be formed [6].

With the help of this rather simple and easily reproducible process, soft but also mechanically rigid networks can be generated. Those networks contain a system of interconnecting micro- and macro-pores ranging between 1 and 200 µm [7,8].

Potential applications of cryogel materials in biomedicine cover numerous biological processes of immobilization, separation, release of active biomolecules, molecular imprinting and sensing, and last but not least, tissue regeneration and engineering [6,7,8,9]. In the early years of cryogel research, the focus of interest was mostly on synthetic polymers [10]. Currently, natural polymers known for their high cell compatibility and hydrophilic properties have received increased attention.

It is the aim of this work to first provide a current overview of the synthesis of glycosaminoglycan (GAG)-based cryogels. Own activities for the preparation of photo-cross-linkable cryogels will also be included. Furthermore, specific features of GAG-containing cryogels will be considered and potential applications of these materials as scaffolds in soft tissue engineering and related fields will be discussed.

## 2. Principles of the Cryogelation Process

**Figure 1:**
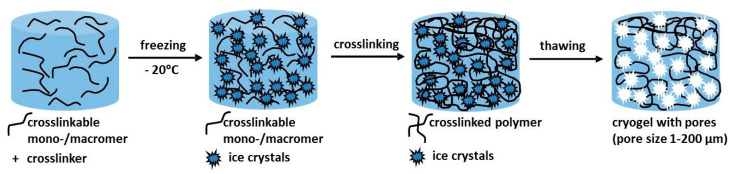
General scheme of the cryogelling process.

In a typical cryogelling process [5,9], an aqueous gel solution is cooled down to −5 to −20 °C. At this temperature range, most of the solvent crystallizes and only a small part remains in the liquid phase. In this liquid phase, dissolved monomers/macromers are concentrated and form physically or chemically linked network structures, mainly through chemical crosslinking. After a suitable period of gelation, i.e., crosslinking reaction, the mixture is thawed to room temperature, forming the three-dimensional, porous, and highly interconnective architecture of the cryogel. The whole process is schematically shown in Figure 1.

Since the procedure usually takes place in water or aqueous solution, there is no need for time-consuming, complete removal of porogens. This is a major advantage since used porogens are often incompatible or even cytotoxic for cells. Compared to conventional hydrogels, which have long been discussed as carrier materials for soft tissue engineering, cryogels have the advantage that the macroporous interconnecting structure is an essential prerequisite to facilitate angiogenesis or to enable vascularization from endothelial precursor cells, their supply with nutrients, and the removal of waste products from the cells [7,9].

Although the production of cryogels is a rather simple technology requiring comparatively little time and effort, there are numerous parameters that determine the physical and, consequently, the related biological properties [9,11]. These variables include the gel composition (type of cross-linkable educts, concentration), the type, degree, and rate of crosslinking, the temperature of gelation, and the rate of freezing [6].

Natural polymers, including polysaccharides such as agarose, alginate, chitosan, and dextran, as well as proteins such as gelatin, collagen, or silk, have already been widely used in cryogel production for biomedical applications due to their excellent biocompatibility with eliciting minimal immune response [6,7,8,9].

## 3. Overview of GAG-Based Cryogels

With regard to applications as cell matrices or scaffolds in tissue engineering and regeneration, highly porous cryogels containing inter-connective macropores with diameters mostly above 50 µm are of special interest. Further essential requirements of cell scaffolds are a high cytocompatibility of the cryolgels, a sufficient mechanical stability or at least mechanical integrity, the possibility to sterilize the scaffold prior to use, and for most applications, also a controlled degradability over time.

Glycosaminoglycans (GAGs) are a family of natural, negatively charged unbranched heteropolysaccharides composed of disaccharide repeating units. Glycosaminoglycans (GAG) and many of their derivatives are known for their innate cytocompatibility. Besides other macromolecules such as proteins, GAGs are part of the extracellular matrix (ECM) providing structural and biochemical support to the embedded and surrounding cells. Structurally, the ECM forms a three-dimensional network of nm-sized protein fibers, mainly based on collagens and elastin, that are surrounded by and embedded in a hydrogel mainly consisting of GAGs and proteoglycans, a group of protein-linked GAGs [12]. As three-dimensional polymeric scaffolds, GAG-based cryogels can perfectly mimic the ECMs of different tissues or at least their gel-like basic hydrogel “ground substance”.

**Figure 2:**
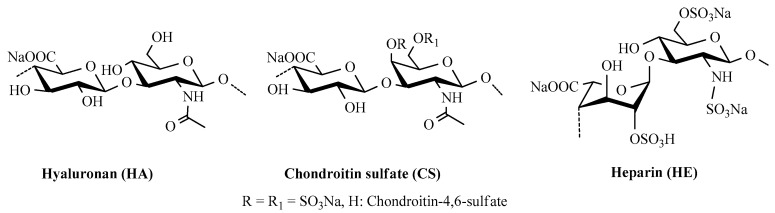
Chemical structures of GAGs used for the preparation of cryogels.

The entire GAG family includes hyaluronan (HA) as the only non-sulfated member, as well as chondroitin sulfate (CS), dermatan sulfate, keratin sulfate, heparan sulfate, and heparin (HE). Due to the limited accessibility of individual GAG members, only cryogels of HA, CS, and HE have been described so far, which will be discussed in more detail below (see Figure 2 for chemical structures).

Due to the polyfunctional character of GAGs, they have numerous functional and easily modifiable groups at their polymer backbone for the introduction of cross-linkable and cell adherence and cell proliferation stimulating moieties. A further advantage of GAGs is their hydrophilic character, allowing them to absorb high volumes of tissue fluids and facilitate cell penetration. Consequently, numerous cryogels based on different GAGs or GAG-derivatives have been described in recent years for applications as polymeric scaffold materials. An overview of different cryogels prepared on the basis of HA, CS, and HE and their derivatives, respectively, is provided in Table 1 and Table 2.

## 4. Preparation of GAG-Based Cryogels

### 4.1. Physical Crosslinking

GAG are typical polyanions enabling polyelectrolyte complex (PEC) formation with polycations such as chitosan or other cationic amino-group-containing synthetic polymers. Although physical crosslinking normally results in rather weak interactions, the described physical crosslinking between polymeric HA and chitosan chains leads to a dimensionally stable construct even after one week of incubation [22]. Physical crosslinking is also known for other ionic polysaccharides such as pectin [51], and even non-charged polysaccharides such as cellulose [52].

### 4.2. Chemical Crosslinking

Due to the known disadvantages of physical crosslinking, such as poor mechanical stability, difficult adjustability of the pore size, and the often-rapid degradation behavior, many researchers use chemical methods of crosslinking [53,54]. Several common low-molecular crosslinking agents such as diepoxides [15], glutar dialdehyde [23,37,49], divinylsulfone [17], or water-soluble carbodiimides (e.g., 1-ethyl-3-(3-dimethylaminopropyl) carbodiimide) [18,19,20,35,36], mostly in the presence of N-Hydroxysulfosuccinimide (Sulfo-NHS) [40,41,42,43,44,45,46,47,48], have also been used for the preparation of GAG-based cryogels. Besides those low-molecular-weight crosslinkers, also oligomeric bifunctional crosslinkers, for example a polycaprolactone-based diisocyanate (PCl-di-NCO) and poly(ethylene glycol)bis-maleimide (PEG-bis(maleimide)), have been used to initiate crosslinking by urethane formation [23] and a Diels−Alder click-crosslinked reaction with a furan-modified HA [33,34], respectively.

**Figure 3:**
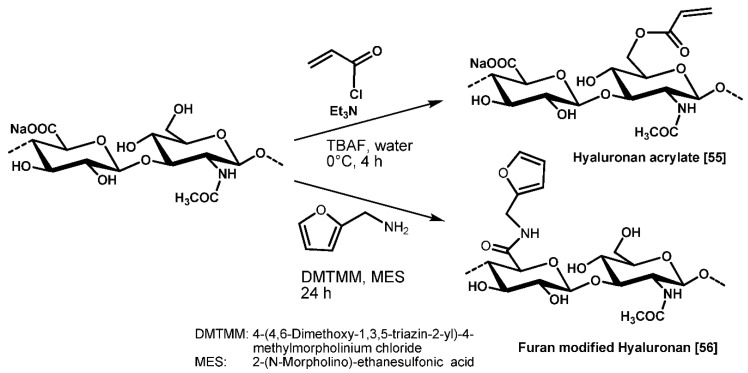
Introduction of reactive functional groups into GAGs, shown exemplarily with HA.

Due to their polyfunctional character, the introduction of reactive, network-forming groups such as acrylic or methacrylic moieties into the different GAG molecules is a rather simple modification reaction, providing (macro)monomers able to undergo free radical polymerization reactions, yielding crosslinked derivatives. Figure 3 shows an example for the introduction of reactive functional groups into the HA backbone. In the upper reaction, acrylate groups are used, which are able to initiate a free radical polymerization reaction [55], and in the lower reaction, furan groups are inserted, able to undergo a Diels–Alder reaction with activated dienophils [33,56].

The free radical polymerization is a three-step process covering an initiation step, the propagation (increasing the polymeric chain length), and termination (ceasing the reactive intermediate in the chain propagation step) within the nonfrozen liquid phase surrounding ice crystals. In a classical way, the polymerization is initiated by adding a radical-forming substance such as a peroxide or an azo compound. To promote an efficient radical formation, normally, temperatures above room temperature are required.

In the case of cryogelation at sub-zero temperatures, promoters such as tertiary amines have to be added to accelerate radical formation. A commonly used initiation system is ammonium persulfate/N,N,N′,N′-tetra-methylethylenediamine (APS/TEMED) [57,58], often also used in GAG cryogelation [27,28,29,30,38,39,50]. Problems with this approach are that the reaction may start as soon as the initiator is added to the solution, which may result in inhomogeneous cryogel structures. The used amine promoter may also cause cytotoxic effects and has to be completely removed from the final product. The use of electron-beam irradiation to initiate radical formation is another method avoiding the use of radical-forming chemicals. The preparation of cryogels based on synthetic polymers [59], but also on polysaccharide- [60] and GAG-methacrylates [24,25,26], by this fast and efficient method has been reported. Photoinitiated free radical polymerization using UV (200–400 nm) or visible (400–800 nm) light is another option to crosslink (meth)acrylated polymers and is often used in the preparations of tissue engineering scaffolds [61,62]. In recent years, considerable work has been investigated in the development of water-soluble, non-toxic photo-initiators [63].

### 4.3. Specific Structural Features of GAG-Based Cryogels

**Figure 4:**
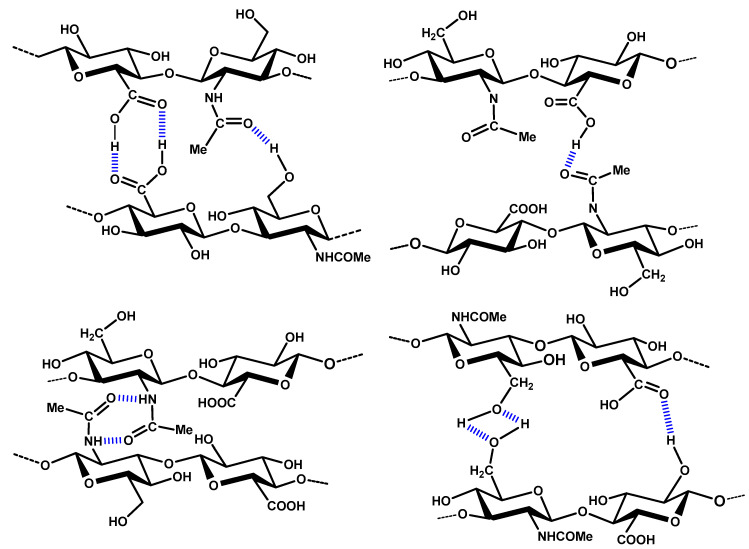
Possible forms of hydrogen bonds between -OH, -COOH, and NHCOCH_3_ groups exemplified by hyaluronan gels (scheme modified according to [64]).

With regard to the polyfunctional character of GAGs with different functional groups (especially OH-, NHCOCH_3_-, COOH-, SO_3_-groups), a three-dimensional network is formed by hydrogen bonding, contributing to a mechanical stabilization of the formed cryogel networks [64] (for possible formations of hydrogen bonds in HA, see Figure 4). Proper adjustment of the pH value of the aqueous solution during the cryogel preparation is therefore an important fabrication parameter.

There exist several pathways for cryogel functionalization: (i) by direct synthesis from monomers or prepolymers, (ii) by introducing functional groups into cryogels after their formation, or (iii) by fabrication of composite cryogels containing different co-comonomers and/or nanofillers [65]. In terms of GAG-containing cryogels, they need the generation of hydrophilic and highly functional networks that are particularly compatible with living cells and have a strong ability to interact with biomolecules, especially with peptides and proteins.

As already summarized by Gun’ko et al. [66], cryogelation from polymers dissolved in water is based particularly on the effect that the freezing water forms pure ice crystallites and results in a more concentrated phase containing the organic components, that does not freeze up to −30 °C. Polymerization in this phase forms the walls of the cryogel, and the amount of water in the crystallites determines the porosity, ranging from nanopores (<0.1 µm) over micropores to macropores (>100 µm) [66]. Due to the presence of interconnected macropores, full hydration is achieved rather quickly in sponge-like cryogels compared to non-porous hydrogels. After complete hydration, the pores of a cryogel become rounded, despite the sharp-edged nature of the solvent crystals that formed them, due to surface tension at the liquid/pore wall interface [1].

Compared to other techniques providing porous scaffold materials, including salt/particle leaching [67], phase separation processes [68], gas foaming [69], or solid freeform fabrication [70], cryogelation tends to be a time- and resource-efficient method.

### 4.4. Fabrication Aspects

**Figure 5:**
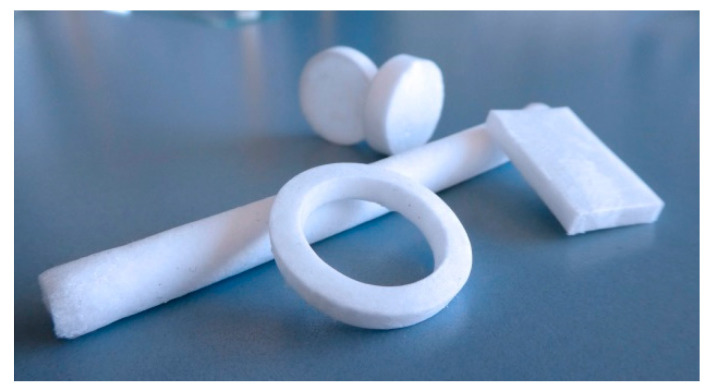
Different geometries of prepared cryogels.

From a technical point of view, depending on the gel preform, cryogels can be produced in different sizes, shapes, and dimensions, as shown in Figure 5. However, fabrication of a continuous structure of very large shape by the cryogelation process is rather difficult to achieve, and the problem of limited control over the morphology of large cryogel structures exists [71]. Furthermore, cryogel mixtures can also be used as coatings for implant devices such as prosthetic grafts to deliver biologics combined with an antithrombotic agent [50].

## 5. Adjustable Application Properties of GAG-Based Cryogels

A number of different process parameters in cryogel synthesis can be easily controlled with regard to the desired properties of the resulting cryogel materials. The most important adjustable parameters and their impact on cryogel features and properties are summarized in Table 3.

### 5.1. Porosity and Interconnectivity

For cell culture materials, pore sizes in the range of 50–200 µm are advantageous. By varying the freezing temperature and thawing process as well as the concentration of the starting materials, the pore size and density can be controlled in a targeted manner.

**Figure 6:**
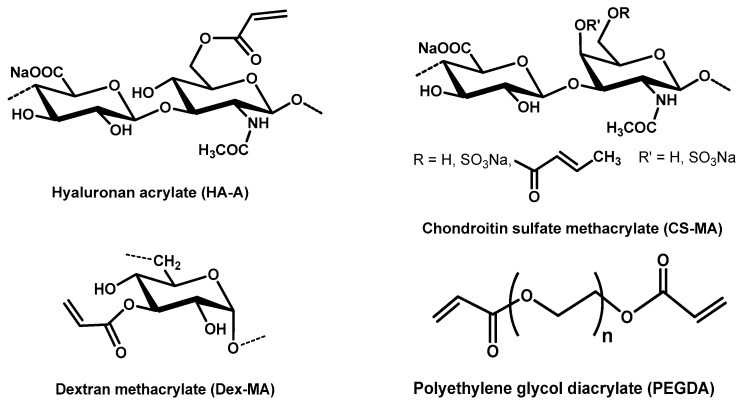
Chemical structures of GAG, polysaccharide (dextran), and crosslinker components used for cryogel preparations.

In our own work, we synthesized various cryogels from acrylated HA (HA-A), methacrylated CS (CS-MA), and methacrylated dextran (Dex-MA), as well as mixtures of HA-A and Dex-MA with polyethylene glycol diacrylate (PEGDA) and CS-MA with Dex-MA (for chemical structure, see Figure 6). Pure PEGDA cryogels were prepared in comparison. We used LAP (lithium phenyl-2,4,6-trimethyl-benzoyl-phosphinate) for crosslinking, which is a non-toxic, water-soluble, easy-to-use photo-initiator in the UV and also visible light range [87]. Cryogels with a diameter of 1.2 cm and a height of 1 cm were manufactured.

The values for the pure density and open and closed cell content of the different prepared cryogels have been measured using a gas pycnometer. Results are provided in Table 4.

The percentage of closed cell content was between 3% and 10% for all cryogels with a starting material concentration of 10% *w*/*w*, whereas the percentage of open cell content was correspondingly at 90–97%. A higher initial concentration of the same material led to a higher pure density and a higher proportion of closed cell content. The pure density of the cryogels varied between 1.1 and 2.4 g/cm³, with the biopolymers Dex-MA and CS-MA clearly being the highest. The largest proportion of open cell content was found in the HA-A gel, as the initial concentration was lowest here at 2.5% *w*/*w*. Theoretically, the proportion of closed cell content in the gel made of 20% *w*/*w* CS-MA should be higher than in the gels with 10% *w*/*w* material concentration. However, the geometric volume of these CS-MA gels was significantly larger than the volume of the other gels, despite the same production process. This might have led to the loosening of the pores and thus to a lower proportion of closed cells. These gels also showed the highest swelling values.

**Figure 7:**
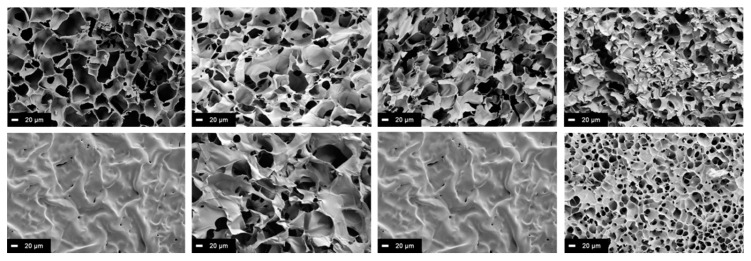
SEM images of different prepared cryogels. The upper row shows the fracture surfaces, the lower row the surfaces. From left to right: PEGDA = polyethylene glycol diacrylate cryogel; HA-A = hyaluronic acid acrylate cryogel; Dex-MA = dextran methacrylate cryogel; CS-MA = chondroitin sulfate methacrylate cryogel.

Using scanning electron microscopy, the morphology of the cryogels can be visualized and the mean pore size estimated (see Figure 7). It was found that the internal pore structure of the individual gels varied greatly. For example, pure PEGDA gels showed symmetrical pores in the interior in the range of 20–80 µm. On the surface, however, only small pores of 10–20 µm were visible. In contrast, the pore sizes of pure biopolymer cryogels were difficult to be determined because the gels were compressed during cutting. Figure 7 shows that the cryogel surfaces of PEGDA and Dex-MA contained only a few small pores. In contrast, HA-A and CS-MA gel surfaces were as porous as the fracture surfaces and had a sponge-like basic structure with pore sizes of 10–60 µm.

### 5.2. Mechanical Properties

The mechanical properties of cryogels are mainly influenced by parameters such as the degree of crosslinking, the porosity/pore size, and the use of additives to fabricate composite cryogels. With regard to conventional hydrogels of comparable composition, cryogels often exhibit superior mechanical properties [8]. For applications requiring higher mechanical resilience or improved elasticity, it is possible to prepare polymeric cryogel blends from various GAG and other natural or synthetic polymers [39,88]. This is especially important for injectable cryogels. Furthermore, hybrid cryogels can also be generated from GAG and nanoscopic fillers [16,48].

**Figure 8:**
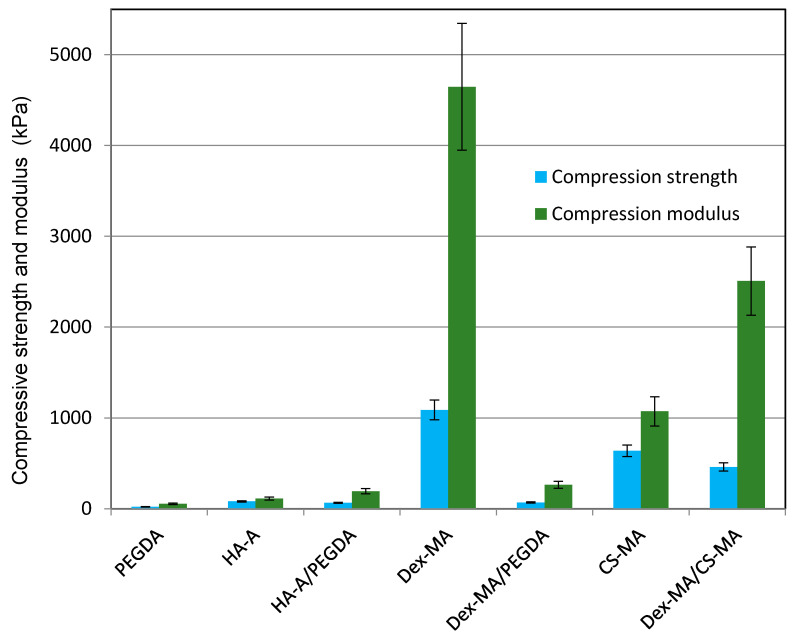
Mechanical strength at 50% deformation and compression modules of GAG-based cryogels in comparison to polysaccharide and synthetic polymer-based cryogels are shown. PEGDA = polyethylene glycol diacrylate; HA-A = hyaluronan acrylate; Dex-MA = dextran methacrylate; CS-MA = chondroitin sulfate methacrylate.

In Figure 8, the compressive strength at 50% deformation of cryogel cylinders prepared from different GAG and dextran compositions respectively (see Table 4), has been determined. It was confirmed that cryogels with higher crosslinking density (higher DS) or higher monomer concentration also showed higher mechanical strength. By adding PEGDA, the pure biopolymer gels became softer and partially showed shape memory properties. Depending on the starting polymer, the compressive strengths were in the range of 20–1300 kPa, with compression modules of 30 to 4700 kPa. The greatest compressive stresses as well as Young’s moduli of freshly produced cryogels were found with Dex-MA.

### 5.3. Anisotropic Morphology

**Figure 9:**
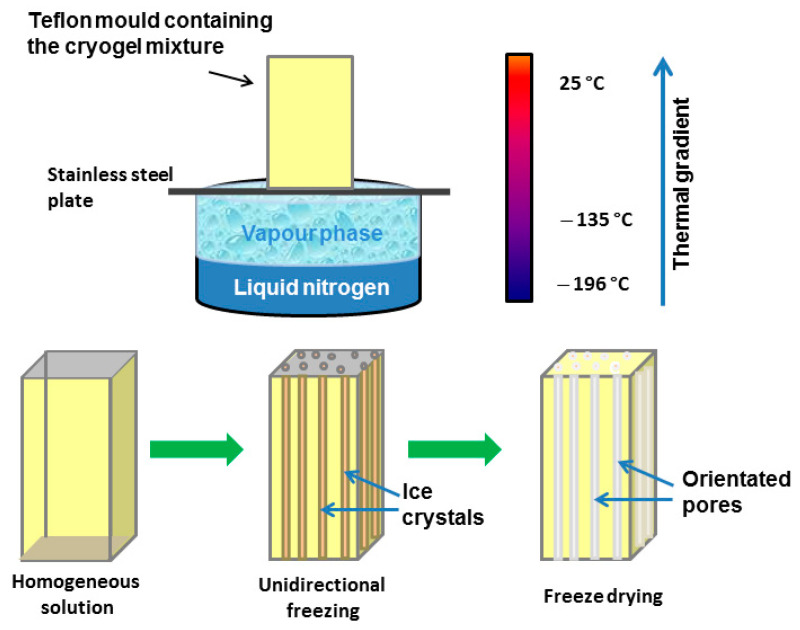
Process scheme for the fabrication of biopolymer scaffolds containing an oriented pore system by unidirectional freezing (scheme modified according to [37] with permission of Elsevier).

Numerous biological gel-like tissues possess an anisotropic hierarchical morphology, resulting in extraordinary mechanical properties. In contrast, those isotropic morphologies can only hardly be mimicked by synthetic hydrogel materials. During recent years, several strategies, including ionotropic gelation [89], 3D-bioprinting [90], electrospinning [91], and even unidirectional freezing [37,92], have been presented to prepare polysaccharide-based cryogels with anisotropic properties. A custom-designed device showing the fabrication process of biopolymer scaffolds with an oriented pore system by unidirectional freezing is shown in Figure 9. It has to be mentioned that the unidirectional freezing process can be used not only for biopolymers such as proteins, various polysaccharides, and GAGs, but also for different synthetic polymers such as waterborne polyurethanes [93].

### 5.4. Stimuli-Responsive Behavior

Another attractive feature already known from hydrogels is the stimuli-responsive behavior of cryogels, which can be initiated by an external physical or chemical trigger such as biological stimuli (e.g., antigens, ligands, enzymes, or small-molecule concentrations such as glucose, nucleic acids), changes in the environment (pH, ionic strength, or molecular species), or physical signals such as temperature, pressure, light, electric, or magnetic fields [94,95,96]. Bond cleavage, bond formation, swelling/deswelling, and conformational changes are common responses of such gels. In recent years, there has been a growing interest for stimuli-responsive cryogels based on polysaccharides and also GAGs for the development of drug delivery systems [97] and scaffolds for tissue engineering [8,71].

## 6. Applications of GAG-Based Cryogels as Scaffolds in Cell Culture and Tissue Engineering

The compositional and structural similarity of GAG-based cryogels to the native ECM combined with their excellent biocompatibility of GAGs and most GAG derivatives make cryogels promising scaffold materials for tissue regeneration and engineering. Previous sections of this manuscript have already referred to relevant reviews. Despite the multitude of work on cryogels, we would like to highlight here the major fields of application and the specific advantages of GAG-based cryogels as scaffolds in cell cultivation and tissue engineering. It should be mentioned that in numerous described applications, composite cryogels made of GAG components with protein-based materials are used [98,99,100].

### 6.1. Cell Culture

It was recently demonstrated by the group of Bencherif [28] that cryogels based on hyaluronan (HA) and gelatin provide a mechanically robust, cell-responsive, macroporous, and injectable platform for the cultivation of various cell types. In this study, fibroblasts and bone marrow-derived dendritic cells have been used. In addition to the known GAG properties, a remarkable advantage of the cryogel system lays in the injectability of a cuboid-shaped cryogel material through a conventional 16-gauge needle. After injection, the deformed cryogel returned to its original shape and surrounding water was reabsorbed into the gel [28]. HA-*co*-gelatin cryogels have also been used for the cultivation of chondrocytes and human mesenchymal stem cells (hMSCs) respectively [27], and also of specific epithelial cells [101].

Furthermore, cryogels based on HA and heparin (HE) also represent biomimetic ECMs to study cancer cell invasion and cell–cell as well as cell–matrix communications of migration in the context of remodeling of the cancer cell microenvironment [102,103]. This understanding, in combination with drug screening experiments, may support the discovery of more efficacious drug targets [102].

### 6.2. Cartilage Tissue Engineering

Due to the absence of vascularization and its limited self-repair ability, cartilage is an important target for tissue engineering. Cartilage injuries are caused by trauma, aging, congenital diseases, or tumor removal. Osteoarthritis, a common joint disorder, is causing tremendous disruption in the patients’ livelihood and daily activities, with many millions of cases worldwide [8]. Although several strategies in cartilage repair, including autologous chondrocyte implantation [104], are currently successfully employed, some drawbacks such as donor-site morbidity, lack of integration, and unmatched properties of the repaired regions limit their application [8]. Biodegradable scaffolds, especially those composed of ECM-containing components such as GAGs have been continuously investigated for cartilage development due to their unique biophysical and biomechanical properties [105]. Recently, both HA- [19] and chondroitin sulfate (CS)-based [35,36,38,39] cryogel scaffolds for cartilage tissue engineering have been fabricated. The addition of glucosamine into HA-*co*-gelatin cryogels can serve as a biological cue for maintaining the chondrogenic phenotype [19]. In another attempt, chitosan was added to HA/CS-*co*-gelatin scaffolds to enhance the mechanical stability of cryogels under dynamic compression conditions during cultivation [35,36]. Han et al. developed ECM-based macroporous cryogels from either methacrylated chondroitin sulfate (CS-MA) or methacrylated HA (HA-MA) crosslinked with gelatin methacrylate or poly(ethyleneglycol) diacrylates (PEGDA) [38,39]. Applying the methacrylated components, the mechanical stability of the cryogels could be significantly improved. Concerning the PEGDA-crosslinked cryogels, expression of cartilage-related genes and accumulation of respective proteins was observed, and after implantation of the scaffolds in mice, led to the formation of a densely interpenetrating network supporting homogeneous cell distribution. Together with the found cartilage-specific ECM productions, the great potential of these cryogel scaffolds becomes clear [8,39].

### 6.3. Skin Regeneration and Wound Healing

In the skin, GAGs are the main components of the ECM in the epidermal, dermal, and hypodermal layers, mainly responsible for the mechanical strength and resistance against wounding. They act in a complex three-dimensional network with other molecules of the connective tissue such as collagen and elastin [106]. The presence of natural macromolecules such as GAGs and various proteins makes the use of such biopolymers useful for the fabrication of scaffolds in skin engineering and wound healing carriers. Some commercially available skin substitutes such as Hyalomatrix^®^, Hyalofill^®^, or Integra^®^ also contain GAG components, such as HA-ester and CS. During the fabrication of the collagen-CS membrane of Integra^®^, a cryogelation step was also employed to provide the porous structure of this membrane [107].

During recent decades, several cryogel matrices with both HA and heparin (HE) as GAG components have been proposed for skin regeneration using crosslinking methods [15,20,23,40]. (Meth)acrylated HA derivatives [26,27] have also been developed. In the work of Thönes et al. [26], crosslinking was performed by E-beam initiation, omitting the addition of any toxic polymerization initiator or amine accelerator. Sterile, highly elastic scaffolds with adjustable pore size, excellent swelling, and low flow resistance properties have been obtained. Human dermal fibroblasts have been cultivated for at least 28 days throughout the cryogels, finding deposition of their own matrix in the pores. Moreover, key modulators of dermal fibroblasts during wound healing such as TGF-β and PDGF efficiently stimulated the expression of wound healing-relevant genes [26].

### 6.4. Nerve Reconstruction and Tissue Engineering

GAGs and other natural polymers have enjoyed widespread application in neural tissue engineering, supporting neurite outgrowth, differentiation, and proliferation on different substrates [108,109]. HA hydrogels enhance the survival rates and proliferation of neural precursors, holding great promise for peripheral nerve regeneration therapies [110] and therapeutic approaches to the central nerve system [111]. Due to the current limitations of autologous and autologous grafts, including morbidity, neuroma formation, scarring, sensory loss, and pro-inflammatory immune response [8], tissue engineering approaches based on hydrogels, and also cryogels that enable nerve regeneration or replacement at the site of injury, are gaining increasing attention [112].

It is already known that GAG-based hydrogels influence the adhesion and differentiation of neural progenitors, opening a new path for therapies targeting neurodegenerative diseases [108]. From this point of view, recently synthesized conductive hydrogels and cryogels [113,114] also represent a new therapeutic approach.

In recent years, a cryogel platform made of HA [115] and HE [44,45] respectively, and starPEG was created covering several aspects of neural regeneration, spanning from nerve guidance conduits for mediating axonal recovery to cell scaffolds for neural, muscle, or stem cells, up to spatiotemporal release of therapeutic agents to support the recovery of damaged CNS tissue [116].

### 6.5. Further Cryogel Applications in Soft Tissue Regeneration

Due to the constant clinical need for reconstruction of soft tissue defects caused by trauma, burns, and tumor resection, autologous fat tissue flaps and commercially available artificial fillers are a major solution for soft tissue augmentation and reconstruction [117]. Mechanically robust and elastic composite scaffolds based on (meth)acrylated HA, gelatin [36], and PEG, as well as CS/HA-*co*-gelatin-*co*-chitosan [30] components, have been loaded with human white adipocyte progenitor cells and human adipose-derived mesenchymal stromal cells and used in a dynamic cell culture approach.

Degeneration of the intervertebral disc, more precisely the nucleus pulposus (NP), is one of the primary causes of back pain worldwide, increasing along with the increasing average age of the world population [118]. Current solutions most widely performed are an invasive, financially costly spinal fusion surgery, or alternatively, disc replacement, also expensive and often connected with problems in the adjacent vertebrae [118]. Cryogels using synthetic materials such as PVA and gelatin/poloxamer composites are already under investigation. Recent nature-based compositions, for example, HA-*co*-Gelatin cryogels [28,119], offer an injectable, mechanically adjustable, cell-adhesive, and cyto-compatible potential platform for this topic.

### 6.6. Tissue Engineering-Related Drug and Cell Release

#### 6.6.1. Drugs and Bioactive Molecules

While high porosity and interconnectivity is an excellent prerequisite for the controlled release of cells from cryogel scaffolds, the controlled delivery of bioactive molecules and drugs is rather problematic and often results in a very rapid but poorly controllable rate of delivery [8]. The adaptation of the release behavior of cryogels according to the respective application demands requires a specific design. In principle, diffusion, swelling, erosion, and stimulus-controlled release profiles can be realized by modifying the cryolgel structure and/or incorporating further structural elements [8,120,121,122,123,124].

With regard to GAG-based cryogels, several papers reported the controlled release of bioactive molecules [125], such as growth factors (e.g., bone morphogenetic proteins (BMP) [47,49], human epidermal growth factor (EGF) [33], vascular endothelial growth factor (VEGF) [46,48], timely release of VEGF and BMP [125], heparin encapsulated interleukin-13 (IL-13) [43], glial cell line-derived neurotrophic factor (GDNF) [44], and nerve growth factor (NGF) [126]).

#### 6.6.2. Cells

In addition to delivering cell growth- or cell differentiation-promoting bioactive molecules and therapeutics to provide an optimal 3D environment for cell culture, cryogels can also be used to deliver cells [8,122]. Concerning GAG-based scaffolds, HA-*co*-gelatin elastic cryogels have been used as transplantation vehicles to transplant adipose-derived stem cells both in a nude mouse and a porcine model [18]. Recently, HE-*co*-starPEG scaffolds, covalently modified with adhesion peptides, have been used for the housing of pancreatic islets in 3D co-culture, with adherent mesenchymal stromal cells (MSC) as accessory cells to improve islet survival and function [41].

GAG-based cryogels have also been applied in several immunotherapeutic approaches [127,128]. One concept is based on the activation of specific T cells by tumor antigen-presenting dendritic cells. Although insufficient survival and localization of transferred cells often limit the clinical efficacy, immune cells’ transplantation within macroporous cryogels may result in better results [8,129]. Another innovative immunotherapeutic approach uses an organoid by housing human mesenchymal stromal cells, gene-modified for the secretion of an anti-CD33-anti-CD3 bispecific antibody, in a biocompatible HE-*co*-starPEG cryogel scaffold as a transplantable and low invasive therapeutic machinery for the treatment of acute myeloid leukemia [42]. This therapeutic device may result as a promising and safe alternative to the continuous administration of short-lived immuno-agents and paves the way for effective bispecific antibody-based therapeutic strategies for future tumor treatments.

## 7. Concluding Remarks

Cryogels are a class of macroporous hydrogels polymerized at sub-zero temperatures, forming mechanically robust, elastic networks. The focus of this review was on cryogels containing mainly glycosaminoglycans (GAGs) or composites of GAGs and other natural polymers. The cryogels produced in this way largely correspond to the native extracellular matrix (ECM) in terms of both composition and molecular structure. As a result, such networks are characterized by an excellent biocompatibility and a controllable biodegradation behavior. Besides their excellent biological properties, cryogels, in general, have several advantages over traditional hydrogels. A major advantage of cryogels is the adjustability of their property profiles via the choice of manufacturing conditions, such as monomer selection, solution concentration, crosslinking density, temperature, and cooling rate. Due to the multi-functionality of GAGs, there are many possibilities for their functionalization into different monomers, which ultimately lead to cryogels with different properties. Vice versa, the functionalization of GAG molecules represents a challenge for the synthetic chemist with regard to a selective reaction control and high-yield monomer products.

Overall, cryogels have received a great deal of interest in the biomedical field, ranging from their use in bioseparation and molecule fractionation to the bioreactor applications to advanced tumor treatment methods. The unique macroporous architecture of cryogels mimicking the native ECM, combined with their physical, especially mechanical and osmotic stability, their shape memory properties, and the often-possible injectability, make GAG-based cryogels promising candidates for polymeric scaffolds in tissue reconstruction and related drug and cell delivery processes. Most recently, GAG-based cryogels have been developed as vehicles for advanced immunotherapies.

Despite numerous technological advances that have been achieved in cryogel research, important questions remain to be answered that will allow this class of materials to be broadly translated into the clinic. From a chemical point of view, such questions concern the exploration of new selective reaction pathways for the functionalization of GAGs and the identification of a reproducible scale-up technology. From a biological perspective, further studies are required on the influence of pore size, volume, and interconnectivity, as well as mechanical parameters on cell physiology and cell differentiation. In conclusion, cryogels, and in particular the GAG-based ones presented here, are versatile biomaterials with a growing future application potential in the biomedical area.

## Figures and Tables

**Table 1:** Overview on relevant publications using hyaluronan (HA)-based cryogels.

GAG	Further Polymer	Crosslinking	Properties	Applications	References
HA	-	EGDE	Large, interconnected macropores (diameters > 100 µm)	Structure-property study	[13,14]
	Collagen (Col)		Decreased swelling with higher Col portion	Dermal fibroblast cultivation	[15]
HA	Halloysite nanotubes (HNTs, Al_2_Si_2_ O_5_(OH)_4_∙nH_2_O)	Divinyl sulfone	Pores size from 50 to 500 µm. Increase in HNT-content enhances mechanical stability, haemocompatible, promoting cell viability, and proliferation	Cell carrier for mesenchymal stem cells and different cancer cells	[16]
HA	-	EDC	Average pore size 18 to 87 μm (large macropores); wide range of elasticity, porosity > 90%, high extensibility, moderate toughness	Soft tissue engineering	[17]
	Gelatin (Gel)			Adipose tissue engineering	[18]
	Gel (+glucosamine, GlcN)		GlcN affects proliferation, and chondrogenic phenotype	Cartilage tissue engineering	[19]
HA	-	Genipin	Interconnected macropores (~100 µm), elastic, low cytotoxicity	Cell culture scaffold, wound healing	[20]
HA	Chitosan	PEC formation	Highly interconnected pore network, porosity: 87%, average pore size: 77 µm, Young’s modulus: 0.2 MPa (dry state)	Mimic of glioblastoma micro-environment ECM	[21]
		Glutar dialdehyde	Porosity > 90%, mean pore size 150–200 µm, high swelling ratio, highly elastic, cytocompatible	Cell culture scaffold	[22]
HA	Atelocollagen	PCl-di-NCO	Dimensionally stable, elastic, high porosity (>93%), hemocompatible	Wound healing	[23]
HA-methacrylate	-	Electron beam-initiated polymerization	Interconnected pores (~70 µm), mechanically stable	Soft tissue engineering	[24,25]
HA-acrylate			Main pore size 70–120 µm, high elasticity, excellent swelling	Skin regeneration, wound healing	[26]
HA-methacrylate	-/Gel-methacrylate	Free radical polymerization (APS/TEMED)	Maintaining shape for 30 days in vitro and in vivo	Skin sculpting, injectable shape-memorizing filler	[27]
	Gel-methacrylate		Macroporous, injectable, improved cell adhesion of biocomposite	Cell carrier	[28]
	Gel-methacrylate, N, N-dimethylacrylamide		Mechanically robust, high frictional resistance	Biomedical application	[29]
	Gel-methacrylate, 4arm-PEG-acrylate		Mechanically robust, injectable, printable	Adipose tissue engineering	[30]
	Dextran-methacrylate		Mechanically robust, Porosity: 80–93%, pore size: 50–135 µm	Tissue engineering scaffold	[31]
HA-methacrylate	-/Gel-methacrylate	UV-Photo-crosslinking (365 nm, Irgacure 2959)	Macroporous, highly permeable gel structure	Cell encapsulation (chondrocytes, hMSCs)	[32]
HA-furfurylamide	PEG-bis(maleimide) (+mono/disaccharides) (+dyes, bioactive ligands)	Diels-Alder reaction	Mean pore sizes 10–30 µm, optically transparent cryogels, Immobilization of dyes, bioactive molecules	Biomimetic cell culture models with 3D spatial control of cellular response	[33,34]

EDC: 1-ethyl-3-(3-dimethylaminopropyl) carbodiimide; EGDE: ethylene glycol diglycidyl ether; APS/TEMED: ammonium persulfate/N,N,N′,N′-tetramethylethylenediamine.

**Table 2:** Overview on relevant publications using chondroitin sulfate (CS)- and heparin (HE)-based cryogels.

GAG	Further (Co)Polymer	Crosslinking	Properties	Applications	References
CS	HA/Gel	EDC	Open connected pore morphology (diameter: 100–350 µm)	Cartilage tissue engineering	[35]
	HA/Gel/Chitosan		Chitosan incorporation increases elastic modulus (stiffness) and toughness; pore diameter: 100–500 µm; cultivation of chondrocytes from rabbit knee articular cartilage Dynamic cultivation of porcine chondrocytes and adipose-derived stem cells under cyclic loading		[36]
CS	HA/Gel/Chitosan/PVA	Glutar dialdehyde	Unidirectional freeze-drying (pore size: 10–210 µm vertical and 20–160 µm transverse section, respectively), porosity 93–98%	Tracheal scaffold fabrication	[37]
CS-methacrylate	Gel-methacrylate	Free radical polymerization (APS/TEMED)	Inter-connected macroporous structure; pore diameters about 89 µm; compressive modulus about 38 kPa; supports chondrocyte phenotype and cellular distribution; subcutaneous implantation of cell-laden cryogel in mice led to dense deposition of cartilage-specific ECM molecules	Cartilage tissue engineering	[38]
CS-methacrylate	PEG-diacrylate	Free radical polymerization (APS/TEMED)	Formation of penetrating polymer network (IPN); ChS-based cryogel showed elevated elastic modulus compared to HA-based system; pore diameter of about 63 µm	Cartilage tissue engineering	[39]
HE	4arm-PEG-NH_2_	EDC/Sulfo-NHS	Macroporous, interconnective 3D-architecture, pore size ranges between 10 and 80 μm; cryogels behave mechanically comparable to the native ECM of soft tissue, showing locally a high resistance to mechanical stress but low bulk stiffness	Endothelial cell cultivation	[40]
				Carrier for pancreative islets	[41]
				Cancer immunotherapy	[42]
				Cytokine release to the brain	[43]
			stem cell culture; RGD-modification-mediated cell adhesion	Neural cell cultivation	[44,45]
HE	Gel	EDC/Sulfo-NHS	Microporous, interconnective architecture stable against enzymatic degradation; injectable	Neovascularization; cell carrier	[46]
	Gel/Whitlockite		stem cell differentiation	Bone tissue engineering	[47,48]
HE	Chitosan; PVA; Hydroxyapatite (HA)	Glutar dialdehyde	Large continuous interconnected pores, slowly degradable network, with 10% HA mechanically stable for bone implantation	Scaffold for growth factor (e.g., BMP-2) delivery	[49]
HE-methacrylate	Alginate-methacrylate; PEG-acrylate-RGD	APS/TEMED	Interconnected porous structure, increase in shape recovery of coated hybrid grafts, enabling cell adherence and growth	Cryogel coating of prosthetic grafts	[50]

PVA: Poly(vinyl alcohol): PEG: Poly(ethylene glycol); Sulfo-NHS: N-hydroxysulfosuccinimide; RGD: Trimeric cell attachment sequence (Arg-Gly-Asp).

**Table 3:** Adjustable process parameters of cryogel synthesis and their effects on cryogel features [8,9].

Parameter	Effect	References
Polymer content/ polymer molecular weight	Gel solutions of lower molecular weight polymers result in the formation of larger pores compared to gel solutions of larger molecular weight polymers. Solutions of higher polymer concentration give a smaller average pore size.	[72,73,74,75]
Crosslinking	Affecting both the stiffness of the cryogel and also the degree of swelling, which in turn impacts on the elastic and mechanical properties. Physical crosslinking: Normally, cryogels with small pore sizes (<100 µm) are formed, and their mechanical strength is inversely correlated with the thawing rate—takes place during the thawing stage. Chemical crosslinking: Commonly larger pore size (>100 µm), improving mechanical stability—occurs during the storage of the solution at the given temperature.	[8,9,76,77]
Cryo-concentration (reaction constituents)	Decreasing the cryo-concentration lowers the critical concentration required for gelling. Increased cryo-concentration increases elasticity	[78]
Cryogelation temperature	Lowering the cryogelation temperature leads to smaller pores, and to thinner and smaller pore walls	[79,80,81,82]
Cooling rate	If the rate of crosslinking proceeds slower than the solvent crystallization, polymerization will generate cryogels of larger pore size; preparation of aligned pore structures	[46,83,84,85,86]
Charge density	Increasing the charge density results in a decreased pore size	[10]

**Table 4:** Comparison of pure density and open and closed cell content of different types of cryogels.

Cryogel Type	Cryogel Concentration (% *w*/*w*)	Pure Density (g/cm³)	Open Cell Content (%)	Closed Cell Content (%)
PEGDA	10	1.14	90.20	9.80
HA-A (DS_A_ ^1^ = 0.2)	2.5	1.49	97.04	2.96
HA-A/PEGDA (1:4)	10	1.91	93.67	6.33
Dex-MA (DS_MA_ ^1^ = 0.5)	10	2.37	94.81	5.19
Dex-MA/PEGDA (1:4)	10	1.82	92.95	7.05
CS-MA (DS_MA_ ^1^ = 0.5)	20	2.43	95.64	4.36
CS-MA/Dex-MA (1:1)	10	2.2	95.69	4.31

^1^ DS_A_, DS_MA_: Average degree of substitution (DS) with acrylate (DS_A_) or methacrylate (DS_MA_) groups related to an anhydrosugar repeating unit.

## Data Availability

The data presented in this study are available on request from the corresponding authors.

## References

[1] Lozinsky V.I. (2002). Cryogels on the basis of natural and synthetic polymers: Preparation, properties and application. Russ. Chem. Rev..

[2] Lozinsky V.I., Galaev I.Y., Plieva F.M., Savina I.N., Jungvid H., Mattiasson B. (2003). Polymeric cryogels as promising materials of biotechnological interest. Trends Biotechnol..

[3] Ashok Kumar A., Mishra R., Reinwald Y., Bhat S. (2010). Cryogels: Freezing unveiled by thawing. Mater. Today.

[4] Gunko V.M., Savina I.N., Mikhalovsky S.V. (2013). Cryogels: Morphological, structural and adsorption characterisation. Adv. Colloid Interf. Sci..

[5] Razavi M., Qiao Y., Thakor A.S. (2019). Three-dimensional cryogels for biomedical applications. J. Biomed. Mater. Res..

[6] Bakhshpour M., Idil N., Perçin I., Denizli A. (2019). Biomedical Applications of Polymeric Cryogels. Appl. Sci..

[7] Hixon K.R., Lu T., Sell S.A. (2017). A comprehensive review of cryogels and their roles in tissue engineering applications. Acta Biomater..

[8] Memic A., Colombani T., Eggermont L.J., Rezaeeyazdi M., Steingold J., Rogers Z.J., Navare K.J., Mohammed H.S., Bencherif S.A. (2019). Latest Advances in Cryogel Technology for Biomedical Applications. Adv. Therap..

[9] Henderson T.M.A., Ladewig K., Haylock D.N., McLean K.M., O’Connor A.J. (2013). Cryogels for biomedical applications. J. Mater. Chem. B.

[10] Okay O., Lozinsky V.I., Okay O. (2014). Synthesis and structure-property relationships of cryogels. Polymeric Cryogel.

[11] Frantz C., Stewart K.M., Weaver V.M. (2010). The extracellular matrix at a glance. J. Cell Sci..

[12] Raina D.B., Kumar A., Kumar A. (2016). Cryogels and Related Research—A Glance over the Past Few Decades. Supermacropotous Cryogels: Biomedical and Biotechnological Applications.

[13] Ström A., Larsson A., Okay O. (2015). Preparation and physical properties of hyaluronic acid-based cryogels. J. Appl. Polym. Sci..

[14] Oelschlaeger C., Bossler F., Willenbacher N. (2016). Synthesis, structural and micromechanical properties of 3D hyaluronic acid-based cryogel scaffolds. Biomacromolecules.

[15] Roether J., Bertels S., Oelschlaeger C., Bastmeyer M., Willenbacher N. (2018). Microstructure, local viscoelasticity and cell culture suitability of 3D hybrid HA/collagen scaffolds. PLoS ONE.

[16] Suner S.S., Demirci S., Yetiskin B., Fakhrullin R., Naumenko E., Okay O., Ayyala R.S., Sahiner N. (2019). Cryogel composites based on hyaluronic acid and halloysite nanotubes as scaffold for tissue engineering. Int. J. Biol. Macromol..

[17] Henderson T.M.A., Ladewig K., Haylock D., McLean K.M., O’Connor A.J. (2015). Formation and characterisation of a modifiable soft macro-porous hyaluronic acid cryogel platform. J. Biomater. Sci. Polym. Ed..

[18] Chang K.-H., Liao H.-T., Chen J.-P. (2013). Preparation and characterization of gelatin/hyaluronic acid cryogels for adipose tissue engineering: In vitro and in vivo studies. Acta Biomater..

[19] Chen C.-H., Kuo C.-Y., Wang Y.-J., Chen J.-P. (2016). Dual Function of glucosamine in gelatin/hyaluronic acid cryogel to modulate scaffold mechanical properties and to maintain chondrogenic henotype for cartilage tissue engineering. Int. J. Mol. Sci..

[20] Roether J., Oelschlaeger C., Willenbacher N. (2019). Hyaluronic acid cryogels with non-cytotoxic crosslinker genipin. Mater. Lett. X.

[21] Florczyk S.J., Wang K., Jana S., Wood D.L., Sytsma S.K., Sham J., Kievit F.M., Zhang M. (2013). Porous chitosan-hyaluronic acid scaffolds as a mimic of glioblastoma microenvironment ECM. Biomaterials.

[22] Kutlusoy T., Oktay B., Apohan N.K., Mediha A., Süleymanoglu M., Kuruca S.E. (2017). Chitosan-co-Hyaluronic acid porous cryogels and their application in tissue engineering. Int. J. Biol. Macromol..

[23] Simionescu B.C., Neamtu A., Balhui C., Danciu M., Ivanov D., David G. (2013). Macroporous structures based on biodegradable polymers—Candidates for biomedical application. J. Biomed. Mater. Res..

[24] Reichelt S., Becher J., Weisser J., Prager A., Decker U., Möller S., Berg A., Schnabelrauch M. (2014). Biocompatible polysaccharide-based cryogels. Mater. Sci. Eng..

[25] Reichelt S., Naumov S., Knolle W., Prager A., Decker U., Becher J., Weisser J., Schnabelrauch M. (2014). Studies on the formation and characterization of macroporous electron-beam generated hyaluronan cryogels. Radiat. Phys. Chem..

[26] Thönes S., Kutz L.M., Oehmichen S., Becher J., Heymann K., Saalbach A., Knolle W., Schnabelrauch M., Reichelt S., Anderegg U. (2017). New E-beam-initiated hyaluronan acrylate cryogels support growth and matrix deposition by dermal fibroblasts. Int. J. Biol. Macromol..

[27] Cheng L., Ji K., Shih T.-Y., Haddad A., Giatsidis G., Mooney D.J., Orgill D.P., Nabzdyk C.S. (2017). Injectable shape-memorizing three-dimensional hyaluronic acid cryogels for skin sculpting and soft tissue reconstruction. Tissue Eng. Part A.

[28] Rezaeeyazdi M., Colombani T., Memic A., Bencherif S.A. (2018). Injectable hyaluronic acid-co-gelatin cryogels for tissue-engineering applications. Materials.

[29] Tavsanli B., Okay O. (2020). Macroporous methacrylated hyaluronic acid cryogels of high mechanical strength and flow-dependent viscoelasticity. Carbohydr. Polym..

[30] Qi D., Wu S., Kuss M.A., Shi W., Soonkyu Chung S., Paul T., Deegan P.T., Kamenskiy A., Yini He Y., Duan B. (2018). Mechanically robust cryogels with injectability and bioprinting supportability for adipose tissue engineering. Acta Biomater..

[31] Zhou D., Shen S., Yun J., Yao K., Lin D.-Q. (2012). Cryo-copolymerization preparation of dextran-hyaluronate based supermacroporous cryogel scaffolds for tissue engineering applications. Front. Front. Chem. Sci. Eng..

[32] Fan C., Ling Y., Deng W., Xue J., Sun P., Wang D.-A. (2019). A novel cell encapsulatable cryogel (CECG) with macro-porous structures and high permeability: A three-dimensional cell culture scaffold for enhanced cell adhesion and proliferation. Biomed. Mater..

[33] Tam R.Y., Fisher S.A., Baker A.E.G., Shoichet M.S. (2016). Transparent porous polysaccharide cryogels provide biochemically defined, biomimetic matrices for tunable 3D cell culture. Chem. Mater..

[34] Owen S.C., Fisher S.A., Tam R.Y., Nimmo C.M., Shoichet M.S. (2013). Hyaluronic Acid Click Hydrogels Emulate the Extracellular Matrix. Langmuir.

[35] Kuo C.-Y., Chen C.-H., Hsiao C.-Y., Chen J.-P. (2015). Incorporation of chitosan in biomimetic gelatin/chondroitin-6-sulfate/hyaluronan cryogel for cartilage tissue engineering. Carbohydr. Polym..

[36] Chen C.-H., Kuo C.-Y., Chen J.-P. (2018). Effect of Cyclic Dynamic Compressive Loading on Chondrocytes and Adipose-Derived Stem Cells Co-Cultured in Highly Elastic Cryogel Scaffolds. Int. J. Mol. Sci..

[37] Yu X., Qian G., Chen S., Xu D., Zhao X., Du C. (2017). A tracheal scaffold of gelatin-chondroitin sulfate-hyaluronan-polyvinyl alcohol with orientated porous structure. Carbohydr. Polym..

[38] Han M.-E., Kang B.J., Kim S.-H., Kim H.D., Hwang N.S. (2017). Gelatin-based extracellular matrix cryogels for cartilage tissue engineering. J. Ind. Eng. Chem..

[39] Han M.-E., Kim S.-H., Kim H.D., Yim H.-G., Bencherif S.A., Kim T.-I., Hwang N.S. (2016). Extracellular matrix-based cryogels for cartilage tissue engineering. Int. J. Biol. Macromol..

[40] Welzel P.B., Grimmer M., Renneberg C., Naujox L., Zschoche S., Freudenberg U., Werner W. (2012). Macroporous StarPEG-Heparin Cryogels. Biomacromolecules.

[41] Borg D.J., Welzel P.B., Grimmer M., Friedrichs J., Weigelt M., Wilhelm C., Prewitz M., Stißel A., Hommel A., Kurth T. (2016). Macroporous biohybrid cryogels for co-housing pancreatic islets with mesenchymal stromal cells. Acta Biomater..

[42] Aliperta R., Welzel P.B., Bergmann R., Freudenberg U., Berndt N., Feldmann A., Arndt C., Koristka S., Stanzione M., Cartellieri M. (2017). Cryogel-supported stem cell factory for customized sustained release of bispecific antibodies for cancer immunotherapy. Sci. Rep..

[43] Schirmer L., Hoornaert C., Le Blon D., Eigel D., Neto C., Gumbleton M., Welzel P.B., Rosser A.E., Werner C., Ponsaerts P. (2020). Heparin-based, injectable microcarriers for controlled delivery of interleukin-13 to the brain. Biomater. Sci..

[44] Newland B., Welzel P.B., Newland H., Renneberg C., Petr Kolar P., Tsurkan M., Rosser A., Freudenberg U., Werner C. (2015). Tackling cell transplantation anoikis: An injectable, shape memory cryogel microcarrier platform material for stem cell and neuronal cell growth. Small.

[45] Newland B., Ehret F., Hoppe F., Eigel D., Pette D., Newland H., Welzel P.B., Kempermann G., Werner C. (2020). Static and dynamic 3D culture of neural precursor cells on Macroporous cryogel microcarriers. MethodsX.

[46] Kim I., Lee S.S., Bae S., Lee H., Hwang N.S. (2018). Heparin Functionalized Injectable Cryogel with Rapid Shape-Recovery Property for Neovascularization. Biomacromolecules.

[47] Kim S.H.L., Lee S.S., Kim I., Kwon J., Kwon S., Bae T., Hur J., Lee H., Hwang N.S. (2020). Ectopic transient overexpression of OCT-4 facilitates BMP4-induced osteogenic transdifferentiation of human umbilical vein endothelial cells. J. Tissue Eng..

[48] Kim I., Lee S.S., Kim S.H.L., Bae S., Lee H., Hwang N.S. (2019). Osteogenic Effects of VEGF-Overexpressed Human Adipose-Derived Stem Cells with Whitlockite Reinforced Cryogel for Bone Regeneration. Macromol. Biosci..

[49] Sultankulov B., Berillo D., Kauanova S., Mikhalovsky S., Mikhalovska L., Saparov A. (2019). Composite Cryogel with Polyelectrolyte Complexes for Growth Factor Delivery. Pharmaceutics.

[50] Huynh C., Shih T.-Y., Mammoo A., Samant A., Pathan S., Nelson D.W., Ferran C., Mooney D., LoGerfo F., Pradhan-Nabzdyk L. (2019). Delivery of targeted gene therapies using a hybrid cryogel-coated prosthetic vascular graft. Peer J..

[51] Konovalova M.V., Markov P.A., Durnev E.A., Kurek D.V., Popov S.V., Varlamov V.P. (2017). Preparation and biocompatibility evaluation of pectin and chitosan cryogels for biomedical application. J. Biomed. Mater. Res. Pt A.

[52] Ciolacu D., Rudaz C., Vasilescu M., Budtova T. (2016). Physically and chemically cross-linked cellulose cryogels: Structure, properties and application for controlled release. Carbohydr. Polym..

[53] Ghorpade V.S., Chen Y. (2020). Preparation of hydrogels based on natural polymers via chemical reaction and cross-linking. Hydrogels Based on Natural Polymers.

[54] Zhang Y., Huang Y. (2021). Rational Design of Smart Hydrogels for Biomedical Applications. Front. Chem..

[55] Becher J., Möller S., Schnabelrauch M. (2013). Phase transfer-catalyzed synthesis of highly acrylated hyaluronan. Carbohydr. Polym..

[56] Nimmo C.M., Owen S.C., Shoichet M.S. (2011). Diels-Alder Click Cross-Linked Hyaluronic Acid Hydrogels for Tissue Engineering. Biomacromolecules.

[57] Berg A., Peters F., Schnabelrauch M., von Byern J., Grunwald I. (2010). Biodegradable (meth)acrylate-based adhesives for surgical applications. Biological Adhesive Systems: From Nature to Technical and Medical Application.

[58] Hwang Y., Zhang C., Varghese S. (2010). Poly(ethylene glycol) cryogels as potential cell scaffolds: Effect of polymerization conditions on cryogel microstructure and properties. J. Mater. Chem..

[59] Reichelt S., Abe C., Hainich S., Knolle W., Decker U., Prager A., Konieczny R. (2013). Electron-beam derived polymeric cryogels. Soft Matter.

[60] Naumov S., Knolle W., Becher J., Schnabelrauch M., Reichelt S. (2014). Electron-beam generated porous dextran gels: Experimental and quantum chemical studies. Electron-beam generated porous dextran gels: Experimental and quantum chemical studies. Int. J. Radiat. Biol..

[61] Fisher J.P., Dean D., Engel P.S., Mikos A.G. (2001). Photoinitiated Polymerization of Biomaterials. Annu. Rev. Mater. Res..

[62] Ifkovits J.L., Burdick J.A. (2007). Review: Photopolymerizable and Degradable Biomaterials for Tissue Engineering Applications. Tiss. Eng..

[63] Tomal W., Ortyl J. (2020). Water-Soluble Photoinitiators in Biomedical Applications. Polymers.

[64] Luan T., Wu L., Zhang H., Wang Y. (2012). A study on the nature of intermolecular links in the cryotropic weak gels of hyaluronan. Carbohydr. Polym..

[65] Setayeshmehr M., Esfandiari E., Rafieinia M., Hashemibeni B., Taheri-Kafrani A., Ali Samadikuchaksaraei A., Kaplan D.L., Moroni L., Joghataei M.T. (2019). Hybrid and composite scaffolds based on extracellular matrices for cartilage tissue engineering. Tissue Eng. Part B.

[66] Savina I.N., Gun’ko V.M., Turov V.V., Dainiak M., Phillips G.J., Galaev I.Y., Mikhalovsky S.V. (2011). Porous structure and water state in cross-linked polymer and protein cryo-hydrogels. Soft Matter.

[67] Tessmar J.K.V., Holland T.A., Mikos A.G., Ma P.X., Elisseeff J. (2006). Salt Leaching for Polymer Scaffolds: Laboratory-Scale Manufacture of Cell Carriers. Scaffolding in Tissue Engineering.

[68] Chen V.J., Ma P.X., Ma P.X., Elisseeff J. (2006). Polymer Phase Separation. Scaffolding in Tissue Engineering.

[69] Huang Y.-C., Mooney D.J., Ma P.X., Elisseeff J. (2006). Gas Foaming to Fabricate Polymer Scaffolds I Tissue Enginnering. Scaffolding in Tissue Engineering.

[70] Hutmacher D.W., Reis R.L., San Roman J. (2005). Design and Fabrication of Scaffolds via Solid Free-Form Fababrication. Biodegradable Systems in Tissue Engineering and Regenerative Medicine.

[71] Tripathi A., Melo J.S. (2019). Cryostructurization of polymeric systems for developing macroporous cryogel as a foundational framework in bioengineering applications. J. Chem. Sci..

[72] Ivanov R.V., Lozinsky V.I., Noh S.K., Lee Y.R., Han S.S., Lyoo W.S. (2008). Preparation and characterization of polyacrylamide cryogels produced from a high-molecular-weight precursor. II. The influence of the molecular weight of the polymeric precursor. J. Appl. Polym. Sci..

[73] Van Vlierberghe S., Dubruel P., Lippens E., Cornelissen M., Schacht E. (2009). Correlation between Cryogenic Parameters and Physico-Chemical Properties of Porous Gelatin Cryogels. J. Biomater. Sci. Polym. Ed..

[74] Memic M., Rezaeeyazdi M., Villard P., Rogers Z.J., Abudula T., Colombani T., Bencherif S.A. (2020). Effect of Polymer Concentration on Autoclaved Cryogel Properties. Macromol. Mater. Eng..

[75] Singh A., Parvaiz A.S., Das M., Seppälä J., Kumar A. (2019). Aligned Chitosan-Gelatin Cryogel-Filled Polyurethane Nerve Guidance Channel for Neural Tissue Engineering: Fabrication, Characterization, and In Vitro Evaluation. Biomacromolecules.

[76] Parhi R. (2017). Cross-Linked Hydrogel for Pharmaceutical Applications: A Review. Adv. Pharm. Bull..

[77] Bernhardt A., Despang F., Lode A., Demmler A., Hanke T., Gelinsky M. (2009). Proliferation and osteogenic differentiation of human bone marrow stromal cells on alginate–gelatine–hydroxyapatite scaffolds with anisotropic pore structure. J. Tissue Eng. Regen. Med..

[78] Lozinsky V.I., Plieva F.M., Galaev I.Y., Mattiasson B. (2001). The potential of polymeric cryogels in bioseparation. Bioseparation.

[79] Yetiskin B., Okay O. (2017). High-strength silk fibroin scaffolds with anisotropic mechanical properties. Polymer.

[80] Zhang Y., Wang C., Jiang W., Wenqian Zuo W., Han G. (2017). Influence of Stage Cooling Method on Pore Architecture of Biomimetic Alginate Scaffold. Sci. Rep..

[81] Sun B., Wang Z., He Q., Fan W., Cai S. (2017). Porous double network gels with high toughness, high stretchability and fast solvent-absorption. Soft Matter.

[82] Serex L., Braschler T., Filippova A., Rochat A., Béduer A., Bertsch A., Renaud P. (2018). Pore Size Manipulation in 3D Printed Cryogels Enables Selective Cell Seeding. Adv. Mater. Technol..

[83] Ozmen M.M., Dinu M.V., Dragan E.S., Okay O. (2007). Preparation of Macroporous Acrylamide-based Hydrogels: Cryogelation under Isothermal Conditions. J. Macromol. Sci. Pure Appl. Chem..

[84] Kathuria N., Tripathi A., Kar K.K., Kumar A. (2009). Synthesis and characterization of elastic and macroporous chitosan–gelatin cryogels for tissue engineering. Acta Biomater..

[85] He X., Yao K., Shen S., Yun J. (2007). Freezing characteristics of acrylamide-based aqueous solution used for the preparation of supermacroporous cryogels via cryo-copolymerization. Chem. Eng. Sci..

[86] Bodenberger N., Kubiczek D., Abrosimova I., Scharm A., Kipper F., Walther P., Rosenau F. (2016). Evaluation of methods for pore generation and their influence on physio-chemical properties of a protein based hydrogel. Biotechnol. Rep..

[87] Rother S., Galiazzo V.D., Kilian D., Fiebig K.M., Becher J., Moeller S., Hempel U., Schnabelrauch M., Waltenberger J., Scharnweber D. (2017). Hyaluronan/Collagen Hydrogels with Sulfated Hyaluronan for Improved Repair of Vascularized Tissue Tune the Binding of Proteins and Promote Endothelial Cell Growth. Macromol. Biosci..

[88] Boyaci T., Orakdogen N. (2016). Poly(N,N-dimethylaminoethyl methacrylate-co-2-acrylamido-2-methyl- propanosulfonic acid)/Laponite nanocomposite hydrogels and cryogels with improved mechanical strength and rapid dynamic properties. Appl. Clay Sci..

[89] Leong J.-Y., Lam W.-H., Ho K.-W., Voo W.-P., Lee M.F.-X., Lim H.-P., Lim S.-L., Tey B.-T., Poncelet D., Chan E.-S. (2016). Advances in fabricating spherical alginate hydrogels with controlled particle designs by ionotropic gelation as encapsulation systems. Particuology.

[90] Radhakrishnan J., Subramanian A., Krishnan U.M., Sethuraman S. (2017). Injectable and 3D Bioprinted Polysaccharide Hydrogels: From Cartilage to Osteochondral Tissue Engineering. Biomacromolecules.

[91] Jun I., Han H.-S., Edwards J.R., Jeon H. (2018). Electrospun Fibrous Scaffolds for Tissue Engineering: Viewpoints on Architecture and Fabrication. Int. J. Mol. Sci..

[92] Arora A., Kothari A., Katti D.S. (2015). Pore orientation mediated control of mechanical behavior of scaffolds and its application in cartilage-mimetic scaffold design. J. Mech. Behav. Biomed. Mater..

[93] Lin W., Lan W., Wu Y., Zhao D., Wang Y., He X., Li J., Li Z., Luo F., Tan H. (2020). Aligned 3D porous polyurethane scaffolds for biological anisotropic tissue regeneration. Regen. Biomater..

[94] Chiriac A.P., Ghilan A., Neamtu I., Nita L.E., Rusu A.G., Chiriac V.M. (2019). Advancement in the Biomedical Applications of the (Nano)gel Structures Based on Particular Polysaccharides. Macromol. Biosci..

[95] Badeau B.A., DeForest C.A. (2019). Programming Stimuli-Responsive Behavior into Biomaterials. Annu. Rev. Biomed. Eng..

[96] Mohamed M.A., Fallahi A., El-Sokkary A.M.A., Salehi S., Akl M.A., Jafari A., Tamayol A., Fenniri H., Khademhossein A., Andreadis S.T. (2019). Stimuli-responsive hydrogels for manipulation of cell microenvironment: From chemistry to biofabrication technology. Progr. Polym. Sci..

[97] Le N.T.T., Nguyen T.N.Q., Cao V.D., Hoang D.T., Ngo V.C., Hoang Thi T.T. (2019). Recent Progress and Advances of Multi-Stimuli-Responsive Dendrimers in Drug Delivery for Cancer Treatment. Pharmaceutics.

[98] He Y., Wang C., Wang C., Xiao Y., Lin W. (2021). An Overview on Collagen and Gelatin-Based Cryogels: Fabrication, Classification, Properties and Biomedical Applications. Polymers.

[99] Saylan Y., Denizli A. (2019). Supermacroporous Composite Cryogels in Biomedical Applications. Gels.

[100] Savina I.N., Zoughaib M., Yergeshov A.A. (2021). Design and Assessment of Biodegradable Macroporous Cryogels as Advanced Tissue Engineering and Drug Carrying Materials. Gels.

[101] Kao H.-H., Kuo C.-Y., Chen K.-S., Chen J.-P. (2019). Preparation of Gelatin and Gelatin/Hyaluronic Acid Cryogel Scaffolds for the 3D Culture of Mesothelial Cells and Mesothelium Tissue Regeneration. Int. J. Mol. Sci..

[102] Tam R.Y., Smith L.J., Shoichet M.S. (2017). Engineering Cellular Microenvironments with Photo- and Enzymatically Responsive Hydrogels: Toward Biomimetic 3D Cell Culture Models. Acc. Chem. Res..

[103] Bray L.J., Secker C., Murekatete B., Sievers J., Binner M., Welzel P.B., Werner C. (2018). Three-Dimensional In Vitro Hydro- and Cryogel-Based Cell-Culture Models for the Study of Breast-Cancer Metastasis to Bone. Cancers.

[104] Davies R.L., Kuiper N.J. (2019). Regenerative Medicine: A Review of the Evolution of Autologous Chondrocyte Implantation (ACI) Therapy. Bioengineering.

[105] Chircov C., Grumezescu A.M., Bejenaru L.E. (2018). Hyaluronic Acid-Based Scaffolds for Tissue Engineering. Rom. J. Morphol. Embryol..

[106] Varkey M., Ding J., Tredget E.E. (2015). Advances in Skin Substitutes—Potential of Tissue Engineered Skin for Facilitating Anti-Fibrotic Healing. J. Funct. Biomater..

[107] Yannas I.V., Burke J.F., Gordon P.L., Huang C., Rubenstein R.H. (1980). Design of an artificial skin. II. Control of chemical composition. J. Biomed. Mater. Res..

[108] Boni R., Ali A., Shavandi A., Clarkson A.N. (2018). Current and novel polymeric biomaterials for neural tissue engineering. J. Biomed. Sci..

[109] Abatangelo G., Vindigni V., Avruscio G.L., Pandis L., Brun P. (2020). Hyaluronic Acid: Redefining Its Role. Cells.

[110] Thomas R.C., Philip Vu P., Modi S.P., Chung P.E., Landis R.C., Khaing Z.Z., Hardy J.G., Schmidt C.E. (2017). Sacrificial crystal templated hyaluronic acid hydrogels as biomimetic 3D tissue scaffolds for nerve tissue regeneration. ACS Biomater. Sci. Eng..

[111] Liang Y., Walczak P., Bulte J.W.M. (2013). The Survival of Engrafted Neural Stem Cells within Hyaluronic Acid Hydrogels. Biomaterials.

[112] Sensharma P., Madhumathi G., Jayant R.D., Jaiswal A.K. (2017). Biomaterials and cells for neural tissue engineering: Current choices. Mater. Sci. Eng..

[113] Humpolíček P., Radaszkiewicz A.K., Capáková Z., Pacherník J., Bober P., Kašpárková V., Rejmontová P., Lehocký M., Ponížil P., Stejskal J. (2018). Polyaniline cryogels: Biocompatibility of novel conducting macroporous material. Sci. Rep..

[114] Jasenská D., Kašpárková V., Radaszkiewicz K.A., Capáková Z., Pacherník J., Trchová M., Humpolíček P. (2020). Conducting composite films based on chitosan or sodium hyaluronate. Properties and cytocompatibility with human induced pluripotent stem cells. Carbohydr. Polym..

[115] Wu S., Kuss M., Qi D., Hong J., Wang H.-J., Zhang W., Chen S., Ni S., Duan B. (2019). Development of Cryogel-Based Guidance Conduit for Peripheral Nerve Regeneration. ACS Appl. Bio Mater..

[116] Eigel D., Werner C., Newland B. (2021). Cryogel biomaterials for neuroscience applications. Neurochem. Int..

[117] Brett E., Chung N., Leavitt W.T., Momeni A., Longaker M.T., Wan D.C. (2017). A Review of Cell-Based Strategies for Soft Tissue Reconstruction. Tissue Eng. Part B.

[118] Temofeew N.A., Hixon K.R., McBride-Gagyi S.H., Sell S.A. (2017). The fabrication of cryogel scaffolds incorporated with poloxamer 407 for potential use in the regeneration of the nucleus pulposus. J. Mater. Sci. Mater. Med..

[119] Cloyd J.M., Malhotra N.R., Lihui Weng L., Chen W., Mauck R.L., Elliott D.M. (2007). Material properties in unconfined compression of human nucleus pulposus, injectable hyaluronic acid-based hydrogels and tissue engineering scaffolds. Eur. Spine J..

[120] Li J., Mooney D.J. (2016). Designing hydrogels for controlled drug delivery. Designing hydrogels for controlled drug delivery. Nat. Rev. Mater..

[121] Koshy S.T., Zhang D.K.Y., Grolman J.M., Stafford A.G., Mooney D.J. (2018). Injectable nanocomposite cryogels for versatile protein drug delivery. Acta Biomater..

[122] Bakhshpour M., Yavuz H., Denizli A. (2018). Controlled release of mitomycin C from PHEMAH–Cu(II) cryogel membranes. Artific. Cells Nanomed. Biotechnol..

[123] Ho M., Teal C.T., Shoichet M.S. (2019). A hyaluronan/methylcellulose-based hydrogel for local cell and biomolecule delivery to the central nervous system. Brain Res. Bull..

[124] Freudenberg U., Liang Y., Kiick K.L., Werner C. (2016). Glycosaminoglycan-Based Biohybrid Hydrogels: A Sweet and Smart Choice for Multifunctional Biomaterials. Adv. Mater..

[125] Lee S.S., Kim J.H., Jeong J., Kim S.H.L., Koh R.H., Kim I., Bae S., Lee H., Hwang N.S. (2020). Sequential growth factor releasing double cryogel system for enhanced bone regeneration. Biomaterials.

[126] Newland B., Newland H., Lorenzi F., Eigel D., Welzel P.B., Fischer D., Wang W., Freudenberg U., Rosser A., Werner C. (2021). Injectable Glycosaminoglycan-Based Cryogels from Well-Defined Microscale Templates for Local Growth Factor Delivery. ACS Chem. Neurosci..

[127] Weiden J., Tel J., Figdor C. (2018). Synthetic immune niches for cancer immunotherapy. Nat. Rev. Immunol..

[128] Leach D.G., Young S., Hartgerink J.D. (2019). Advances in Immunotherapy Delivery from Implantable and Injectable Biomaterials. Acta Biomater..

[129] Bachmann D., Aliperta R., Bergmann R., Feldmann A., Koristka S., Arndt C., Loff S., Welzel P., Albert S., Kegler A. (2018). Retargeting of UniCAR T cells with an in vivo synthesized target module directed against CD19 positive tumor cells. Oncotarget.

